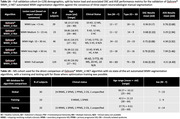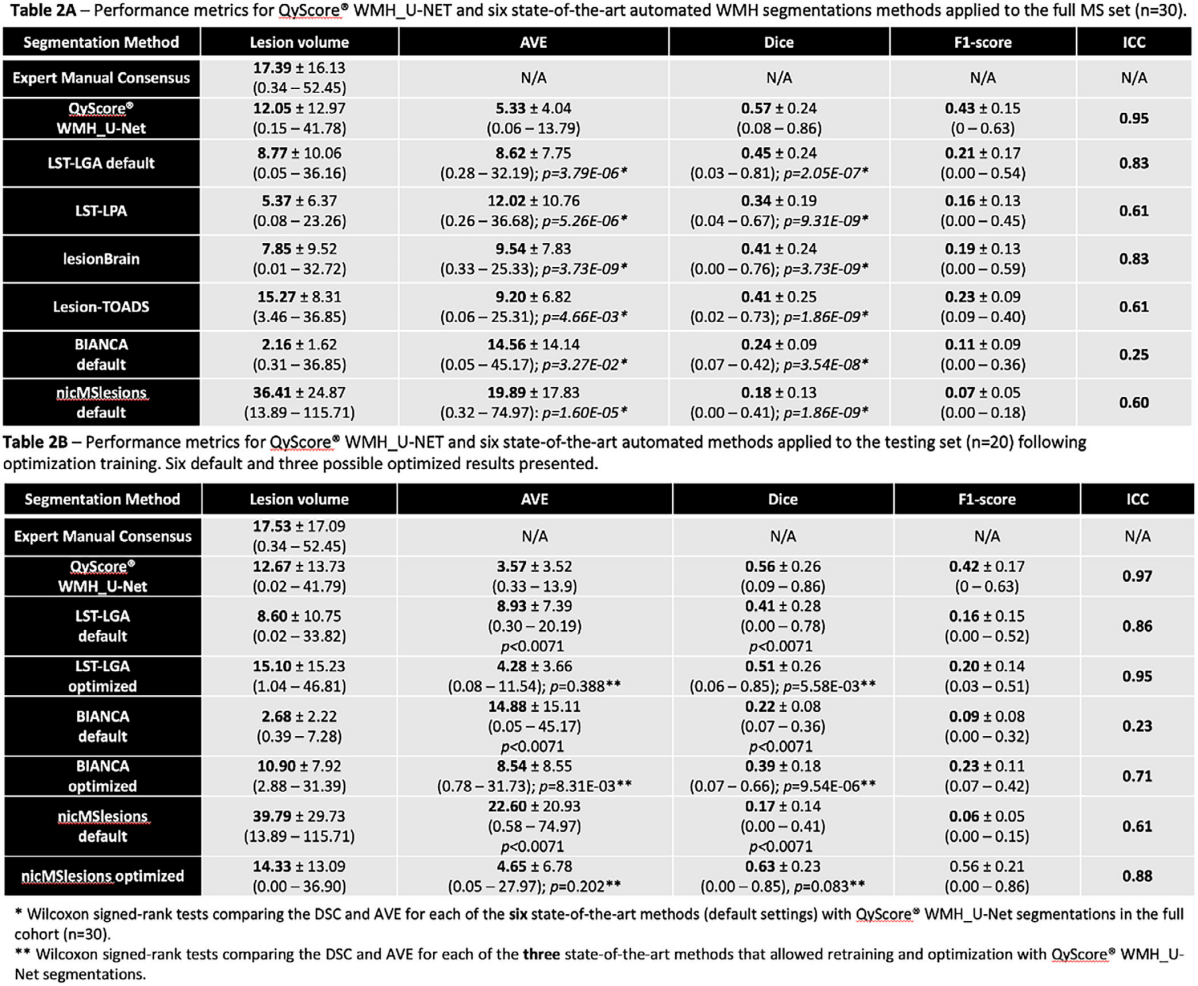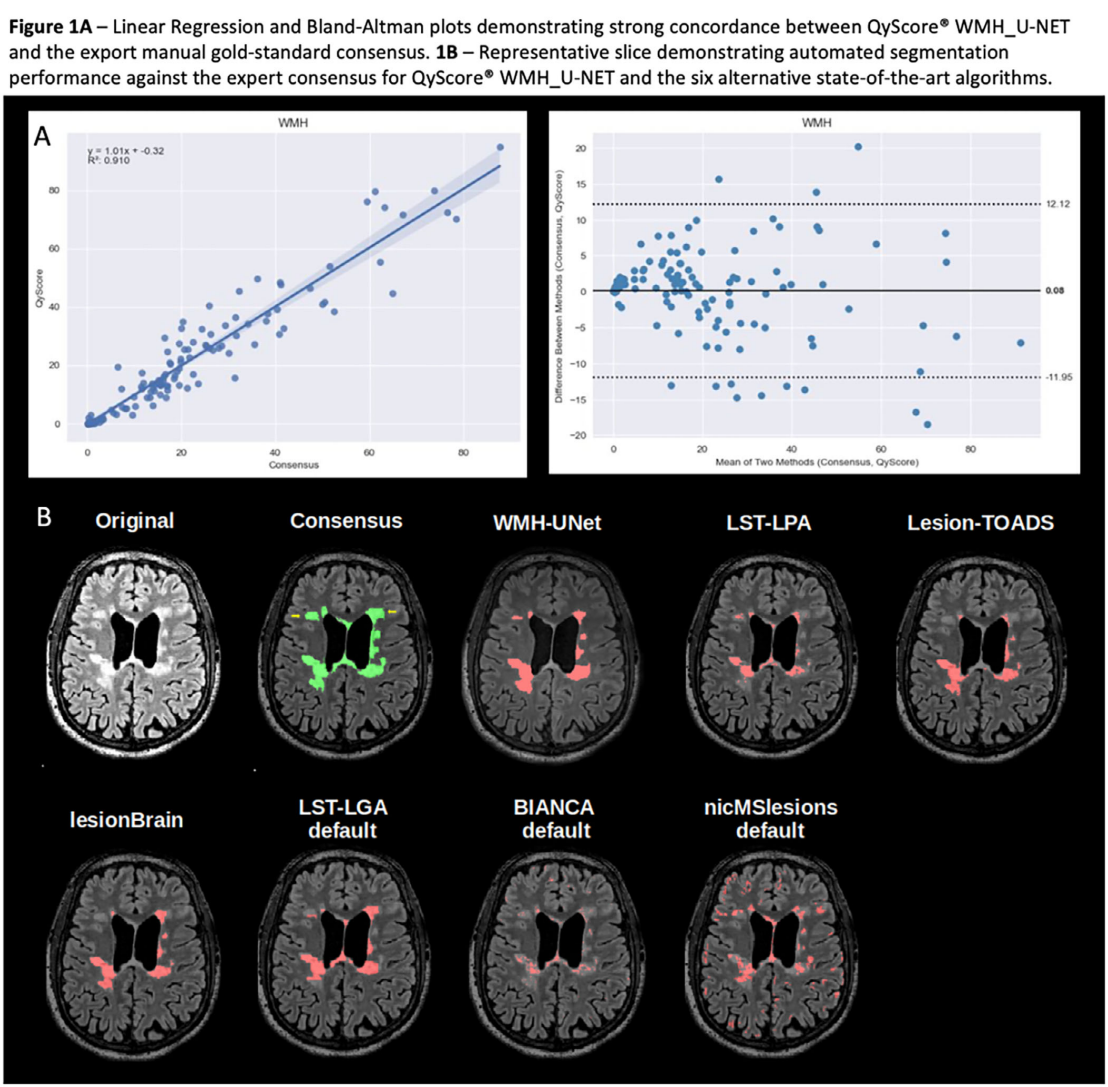# Validation of QyScore® White Matter Hyperintensity (WMH) U‐Net segmentation algorithm against expert manual consensus and comparison with currently available state‐of‐the‐art methods

**DOI:** 10.1002/alz.085347

**Published:** 2025-01-09

**Authors:** Elizabeth Gordon, Luca Villa, Nicolas Guizard, Philippe Tran

**Affiliations:** ^1^ Qynapse, Paris France

## Abstract

**Background:**

Detection and quantification of White Matter Hyperintensities (WMH) are clinically important across multiple CNS disorders and neurodegenerative dementias. However, the prohibitively labor‐intensive nature of manual segmentation limits widespread clinical application. Accurate automated methods for segmenting WMH are needed to overcome this unmet clinical need.

**Method:**

The validation cohort consisted of 129 individuals who had undergone T1‐weighted and T2‐FLAIR MR imaging. To ensure robust results, different scanners (30 GE, 26 Philips, 73 Siemens) and varied patient populations (including Alzheimer’s Disease (n=49), Frontotemporal dementia (n=5) and Multiple Sclerosis (n=45)) were included (Table 1A). The WMH_U‐Net algorithm included in QyScore®, an FDA‐approved and CE‐marked neuroimaging platform, automatically segmented WMH in each image set. These were compared to the gold‐standard consensus of three expert neuroradiologist manual segmentations. Next, a direct comparison of QyScore® WMH_U‐Net was run with six state‐of‐the‐art supervised and unsupervised segmentation methods (LST‐LGA and LPA, Lesion‐TOADS, lesionBrain, BIANCA and nicMSlesions) on a dedicated MS dataset (Table 1B) with default and optimized settings were available. Performance metrics included spatial overlap (Dice Similarity Coefficient (DSC) and F1 scores) and volume comparisons (intra‐class correlation coefficient (ICC) and absolute volume error (AVE, ml)).

**Result:**

QyScore® WMH_U‐Net demonstrated good volume and spatial overlap (average DSC=0.66±0.2), especially with larger WMH load (15‐30ml: DSC=0.75±0.07) across the validation cohort. Optimizing and/or retraining LST‐LGA, BIANCA and nicMSlesions, improved their performances substantially; however, QyScore® WMH_U‐Net remained comparable with the optimized nicMSlesions, with better performance than the optimized LST‐LGA and BIANCA. Friedman test (ANOVA) revealed significantly better spatial (DSC: p=5.90x10^‐21^) and volumetric agreement (AVE: p=3.61x10^‐6^) between QyScore® WMH_U‐Net and the other methods (default settings). Wilcoxon signed‐rank post‐hoc analysis (Bonferroni corrected: p<0.0071) demonstrated QyScore® WMH_U‐Net significantly outperformed all methods for spatial and volumetric agreement (Table 2).

**Conclusion:**

QyScore® WMH_U‐Net produced, robust and the most accurate WMH segmentations across multiple scanners and patient groups, supporting its widespread application for clinical routine practice. It is because it is using a U‐Net approach which is understood to be a fast method for image segmentation over more conventional computer vision methods.